# Is Smiling the Key? Machine Learning Analytics Detect Subtle Patterns in Micro-Expressions of Infants with ASD

**DOI:** 10.3390/jcm10081776

**Published:** 2021-04-19

**Authors:** Gianpaolo Alvari, Cesare Furlanello, Paola Venuti

**Affiliations:** 1 Department of Psychology and Cognitive Sciences, University of Trento, 38068 Rovereto, Italy; paola.venuti@unitn.it; 2 Data Science for Health (DSH) Research Unit, Bruno Kessler Foundation (FBK), 38123 Trento, Italy; 3 HK3 Lab, 38068 Rovereto, Italy; cesare.furlanello@hk3lab.ai; 4 Orobix Life, 24121 Bergamo, Italy

**Keywords:** autism spectrum disorder, facial expressions, machine learning, early detection, ecological

## Abstract

Time is a key factor to consider in Autism Spectrum Disorder. Detecting the condition as early as possible is crucial in terms of treatment success. Despite advances in the literature, it is still difficult to identify early markers able to effectively forecast the manifestation of symptoms. Artificial intelligence (AI) provides effective alternatives for behavior screening. To this end, we investigated facial expressions in 18 autistic and 15 typical infants during their first ecological interactions, between 6 and 12 months of age. We employed Openface, an AI-based software designed to systematically analyze facial micro-movements in images in order to extract the subtle dynamics of Social Smiles in unconstrained Home Videos. Reduced frequency and activation intensity of Social Smiles was computed for children with autism. Machine Learning models enabled us to map facial behavior consistently, exposing early differences hardly detectable by non-expert naked eye. This outcome contributes to enhancing the potential of AI as a supportive tool for the clinical framework.

## 1. Introduction

Autism Spectrum Disorder (ASD) identify a highly complex and pervasive neurodevelopmental condition. It is characterized by a range of symptoms related to socio-communicative abilities and repetitive patterns, restricted behaviors, or interests. Early diagnosis of ASD is a key research and clinical goal. Identifying the presence of the condition as early as possible is crucial to enhance the impact of the treatment, thus directing a vigorous research effort for the identification of behavioral markers that can support the anticipation of a clinical diagnosis [1,2,3,4,5]. Nonetheless, it is still unsolved how to implement an effective screening before the second year of life, in particular, to obtain reliable quantitative measurements of social abilities. Artificial Intelligence (AI) applied to data from optical or other types of sensors offers novel solutions for a systematic and automated analysis of human behaviors. Such tools have already been applied to ASD research to speed up the diagnostic process based on early behavioral markers that emerged from past literature, e.g., reduced attention to social stimuli, reduced response to name, and atypical postural control [6,7,8,9,10,11]. In this study, we aim at exploring AI-based technologies to explore novel Autism Spectrum Disorder (ASD) markers related to facial micro-expressions already present within the first year of development.

### 1.1. Early ASD Detection

The early detection of ASD is an essential objective to improve treatment and long-term quality of life of persons with this condition. From a practical perspective, being able to diagnose the condition as soon as possible offers the opportunity to achieve greater efficacy through specialized intervention pathways and generate more promising prospects for improvement [1,5]. Being able to intervene early during cognitive development, i.e., within the first years of life, would allow exploiting early brain plasticity, anticipating the complete manifestation of the condition, and hopefully containing the atypical drift of cognitive development, at least in part [12]. From a theoretical perspective, understanding ASD early in ontogenesis offers an outlook towards understanding the critical processes for the early socio-communicative maturation at the basis of both atypical and typical development [13]. Despite the growing interest in this direction, succeeding in finding reliable results proved to be complex. Within infancy, manifestations of the condition seem to vary from one individual to another and emerge at different stages of maturation. This pattern reflects both the strong heterogeneous nature of the condition and the variability of early development periods [14,15,16].

Overall, the detection of early prodromal symptoms has proved challenging. There is solid evidence that indices of atypical development can be identified in the second year of life when the deficits in the socio-communicative domain and the presence of stereotyped behaviors are more pronounced [13,17,18,19]. Nevertheless, it is still unclear which ASD manifestations are precocious and specific to the condition. At 12 months of development, differences emerge in infants with ASD related to atypical interactive patterns in the first social exchanges with parents. These behaviors mainly include a reduced capacity of name orientation [20] reduced eye contact [21,22], odd object play [23,24], a decreased responsiveness in episodes of joint attention [22] and a general poor expression [13,15,18,25]. Consistently, when analyzing dyadic synchrony and responsiveness, behavioral differences emerged only from the end of the first year of life among infants with a high risk for ASD [26,27,28]. Specifically, by exploring rates of social smiling, divergences seem to only arise starting at 12 months that widen over time [27,28,29]. Therefore, the second year of development seems to be a critical period within the socio-communicative domain. At this stage, social skills emerge and become key tools for communication that toddlers begin to actively employ. It can be assumed that we can consistently detect deficits at this age in ASD [30]. Nonetheless, detecting the condition in the second year of life may already be too late in terms of maximizing the outcome of the intervention. An important objective is to lower the age threshold for screening as early as possible to anticipate the full manifestation of the phenotype and providing the child with adequate tools for interactive experiences [31,32].

Further, the investigation of signs of atypical behaviors within the first year of development can shed light on the primary components of subsequent symptoms [19]. However, identifying markers can be challenging within the first months of development, when deficits are less evident. At such low ages, atypical behaviors may be subtle and difficult to detect [18,33].

This approach exploits the availability of novel technologies supporting quantitative behavior analysis. For instance, eye-tracking can detect anomalies in the early attentional components of social stimulus processing, showing that infants with ASD are less likely to seek social stimuli and show atypical processing of faces already at 6 months of life [13,30,34,35,36]. In parallel, interesting results emerged from research about motor analysis. Early deficits have been identified related to its primary components, such as symmetry aspects, postural control, and the presence of repetitive movements [25,37,38,39,40]. The integration of these two approaches already holds promising prospects for improving early screening. However, there is limited knowledge about primary components that precede the progressive failure in developing adequate socio-communicative skills in ASD [30]. Social attention seems to play a fundamental role within the definition of the first condition stages, suggesting that these deficits can emerge very early in infants with ASD, leading to a cascade of losses in the development of social competencies. Difficulties in processing incoming social stimuli can make the interactive experience progressively less rewarding, leading to a loss of a toddler’s interest in the surrounding social environment [30,41]. In this perspective, being able to detect the first anomalies in primary components is a priority. In practical terms, to serve as alarm signals, the identification of early pathological signs for the primary components should be (i) based on markers easier to detect, even by non-experts, and possibly (ii) be feasible with the support of technologies.

In summary, identifying ASD earlier means starting treatment earlier and anticipating the overt emergence of social symptoms. Moreover, creating an approach to automated marker identification would enable therapists and caregivers to provide the infant with the necessary tools to properly interact with the environment and gain appropriate experiences while compensating as much as possible for the atypical developmental patterns.

### 1.2. Computer Vision in ASD

Over the last few years, the adoption of AI has increased substantially in human behavior research, achieving promising successes also in the ASD context, where Machine Learning models have the potential to provide enormous support in studying and monitoring symptoms [10]. A first, clear benefit of AI is to offer objective quantitative measurements to categorize behaviors [42]. The application of AI methods for ASD research has led to computer models implemented to speed up the diagnostic process and support the monitoring of deficits such as reduced social attention and atypical postural control [9,10]. Campbell and colleagues [7] proposed the application of Computer Vision (CV) to compare typical and atypical responses in “answer-to-name” tasks by tracking the movements of the head from videos. Differences emerged by comparing typically developing infants and infants with ASD between 16 and 31 months of age, who showed longer latency of response to name [7].

Considering primary motor components, AI-based analysis revealed anomalies in the head movements [8,43]. Toddlers with ASD showed significantly higher rates of head movement compared to typically developing children, suggesting difficulty in holding head position during attentional tasks [8] Moreover, Egger and colleagues [9] developed a mobile-based app to collect data from young children in non-clinical contexts. Infants (12–72 months) were observed while watching short movies designed to elicit autism-related behaviors, and AI algorithms were applied to quantify emotions. Significant differences occurred in affect displays of toddlers with ASD, who showed increased neutral and lower positive expressions compared to controls [9].

Further, Sapiro and colleagues [10] highlighted a high potential of CV models in ASD research to analyze the dynamic nature of infant behaviors during interactions with an accurate continuous measurement. Moreover, AI-based methods provide analyzing behaviors in unconstrained setups, allowing encoding in a more ecological environment, such as the home [44]. In this regard, such tools can be implemented to help non-experts detect and monitor ASD symptoms, paving the way for personalized treatment.

Finally, in a more research-related perspective, AI-based methods can be used to study new indicators of the condition, thanks to a richer characterization of patterns and features that could add to markers already known in ASD literature, such as attentional and motor deficits [7,8,9,43]. CV analysis has already proven to be effective in detecting subtle differences that caregivers and clinicians find hard to recognize with the naked eye [8,42].

### 1.3. Current Study

The current study aims at experimenting with the potential of CV to explore novel markers of ASD from videos. Based on findings in the literature about early deficits in socio-attentive abilities within ASD [30,35,36], we investigated the appearance of Social Smiles [45,46,47] in early ecological interactions among infants with ASD and caregivers. We thus collected retrospective videos of infants between 6 and 12 months of development for a total of more than 3000 usable frames for each subject. Facial behavior was analyzed using the OpenFace AI-based software [48] for refined micro-movements coding, obtaining salient features. The morphological patterns of positive expressions defined by these features were explored for early identification of infants later found to develop ASD. We hypothesized that through a fine-grained analysis of facial dynamics it is possible to detect subtle differences in expressions with communicative value when infants with ASD interact positively with their mothers.

## 2. Methods

### 2.1. Participants

All procedures of our study were in accordance with the ethical standards of the Italian Association of Psychology (AIP), the ethical standards of the Ethics Committee of the APSS (Azienda Provinciale per i Servizi Sanitari, Trento, Italy), and the up-to-date Declaration of Helsinki [49]. The research sample was recruited at the Observation, Diagnosis and Formation Laboratory (ODFLab, University of Trento, Rovereto, Italy), a clinical and research center specialized in the functional diagnosis of neurodevelopmental disorders. The families came to the ODFLab for a detailed profile of child functioning. They were informed about the research study for possible inclusion. After being properly informed about the procedure, they signed informed consent to use anonymized clinical data and provide videotapes of interactions with their child between 6 and 12 months of age. Population characteristics for this study are summarized in Table 1. The study involved video recordings of 18 children with ASD, with an average age of 8 (standard deviation, SD = 1) months. Inclusion criteria for the clinical sample required that children followed a complete clinical evaluation performed by specialists, and the diagnosis was confirmed by ASD as described according to DSM IV/V (Diagnostic and Statistical Manual of Mental Disorders) and ADOS-2 (Autism Diagnostic Observation Schedule) [50] criteria and had no comorbidities with other psychiatric conditions. A sample of video recordings of 15 typically developing (TD) children with an average age of 9 (SD = 2) months was also recruited via public dissemination of the research project from the ODFLab. Inclusion criteria for the control group included that children had no identifiable condition according to DSM IV/V criteria and no significant social development anomaly or family history of neurodevelopmental disorders reported by parents.

Most of the study population was male (ASD 94%, TD 75%), consistent with the asymmetric relationship with sex in ASD pathology [51]. All the children involved in the study were Italian. At the time of data collection, the average age of the participants was 74 (SD = 42) months for ASD and 85 (SD = 30) months for controls. The average ADOS-2 [50] score was 7 (SD = 1.8) for the ASD sample.

A measure of cognitive development was determined by administering the Griffiths Mental Development Scales [52] to 8 children and the Wechsler Intelligence Scale for Children [53] for the rest of the sample. The average Intelligence Quotient, IQ = 76 (SD = 23) was observed over 16 subjects in the ASD group; for two subjects from the ASD group, an IQ score was missing. A measure of cognitive development was not available for the entire TD group, but all children were reported by parents as functioning properly for their chronological age. The average IQ = 96 (SD = 6) was found over 4 subjects in the TD group.

For each of the subjects, Home Videos (HVs) of early interactions between caregivers and the child aged between 6 and 12 months were collected. From the HVs, positive interactions, defined as continuous exchanges between the child and caregiver during playtime, have been extracted (at different ages, 6–12 months). The selection criteria of the interactions required that exchanges were continuous (with no interruptions longer than 5 s) and free of any emission of discomfort or crying, involved the mother, lasted no less than 20 s each, and that the child′s face was frontally visible. Since we wanted to analyze Social and Simple Smiles and non-Social Smiles do not necessarily involve visual coordination, gaze direction was not considered in the inclusion criteria. In this manner, only sequences that met these criteria were extracted and merged from HVs. As a result, video segments of positive interactions were collected for each subject. The average number of interactions selected for each subject was 4 (SD = 1) in the ASD group and 3 (SD = 1) in the TD group. Once merged, a composite video was collected for each subject, with an average duration of 122 (SD = 6) s in the ASD group and 123 (SD = 6) s in the TD group. The HVs were edited for effective analysis of the child’s face by obscuring other faces in the frame and cutting frame sequences in which the child’s face was covered or not frontal.

Finally, by comparing the descriptive variables (Gender, Age, and HVs characteristics), no statistically significant differences emerged between the distributions of the ASD and TD groups (Table 1).

### 2.2. Facial Micro-Expressions

The facial expressions analysis was based on the manual Facial Action Coding System (FACS). Developed by Ekman and Friesen [54], the FACS has been taken as a reference to identify and quantify the observable face micro-movements. As these movements are the expression of the contraction of the underlying muscles, FACS provides a complete set of features that are labeled as Action Units (AUs) to represent the constitutive elements of facial displays, allowing each possible expression to be decoded and measured [54].

Of interest for this study, the FACS describes a set of different types of positive expressions (smiles) linked by the AU12 activation, corresponding to the contraction of the zygomaticus major muscle [54]. We considered a particular smile type, the Social Smile, which is distinguished by the additional activation of AU06, the FACS AU associated with the contraction of the orbicularis oculi muscle [54]. At a behavioral level, social smiling involves the dynamic combination of an expressive (smiling) and an attentive (gazing) component [55]. The Social Smile has been well investigated in literature and has been associated with the morphological conformation of the communicative smile, perceived as more intense and expressive [56,57]. Within the first early interactions between the child and the caregiver, the Social Smile is characterized by being perceived as a more intense expression, compared to Simple Smiles [57,58,59,60], and to occur more frequently in interactive periods in which infant’s attention is directed towards the face of the smiling mother [46,61]. This communicative expression emerges very early in the developmental course and matures within the first semester of life, in conjunction with the acquisition of new visual attention patterns to social stimuli [47,62].

In this study, positive facial expressions with communicative value (Social Smiles, SO) and more neutral expressions (Simple Smiles, SI) were analyzed in the HV dataset described in the previous section, referring to the FACS system for the encoding [54].

### 2.3. Automated Facial Expressions Analysis

The FACS has been widely used in the literature for systematically coding facial expressions. However, it is dependent on time-consuming manual coding of AUs by trained experts. Recently, the development of new AI-based models has allowed the automatic measurement of AUs effectively, highly simplifying the annotation effort [63,64]. Openface automated analysis software for facial behavior was thus included in this study to estimate AUs activation intensity [48].

Openface is based on an architecture trained on multiple datasets of AUs, manually coded by trained experts [48]. Openface has been successfully adopted in several research contexts to analyze AUs patterns in videos [65,66,67]. Besides, it has been tested to map facial dynamics on clinical samples, including autism [68,69,70].

The Openface framework procedure starts with a face detection step, followed by a facial landmark detection analysis. In the final step, face detection and landmarks are processed to compute the AUs for each frame of the video [48]. The initial step is performed by using a multi-task cascaded Convolutional Neural Network (CNN) for face detection and alignment [71]. Afterward, a Convolutional Experts Constrained Local Model (CE-CLM) is implemented for facial landmark detection and tracking in-the-wild scenarios [72]. Consequently, to extract facial appearance features from images, Histograms of Oriented Gradients (HOGs) are applied to the aligned image [73]. A Principal Component Analysis (PCA) model was then implemented to reduce the feature dimensionality [48]. Finally, for the AU presence prediction, a linear kernel Support Vector Machine (SVM) was used; for AU intensity, a linear kernel Support Vector Regression, allowing to analyze their occurrence, co-occurrence, and dynamics [48]. Moreover, Openface contains an implementation that operates better on natural video sequences [74]. The model was trained on several AU datasets available online, then applied to the HV dataset obtaining a collection of 17 AUs. We were able to generate a vector of intensities for each AU in any frame of the video sequence. Then we selected AUs 12 and 6 for quality control and signal processing steps.

### 2.4. Signal Processing

Openface provides a confidence score (C) of the estimations for each frame of the video through a three-layer CNN trained to predict landmark detection error [48]. In this regard, only frames with high internal reliability (C > 0.75) were included in the present analyses [48]. The average percentage of frames included in the analysis exceeds 88%, with an average C of over 0.9 in both samples (Table 1).

The software output was then processed using the pyphisio python library for physiological signal processing [75]. In order to smooth the signal, a convolutional filter with 4-frames convolutions was applied. Consequently, a minimum threshold of duration for the expressive events has been established. Referring to the literature, intensity peaks too short to be considered facial expressions (<1 s) were excluded from the analysis [76,77]. Given this filtered signal, a set of signal processing features from the AUs activation peaks characterizing the interactions were then extracted. Three variables were computed for the extracted peak signals: average duration, the average intensity of AUs, and average frequency (defined by the average number of peaks in 120 s). These variables were extracted for each of the two salient facial expressions: Social Smiles (AU12 and AU06 both active) and Simple Smiles (only AU12 active).

### 2.5. Statistical Analysis

For all dependent variables of duration, frequency, and intensity for the two types of smiles: Social (SO) and Simple (SI), a one-way Multivariate Analysis of Variance (MANOVA) was performed with the groups (ASD vs TD) as an independent factor. Two series of Spearman’s Rank Order Correlations (rho) controlling for IQ scores and sex were calculated within the ASD group to assess the correlations with the dependent variables, to ascertain that results were not related to IQ or gender. Follow-up analysis included one-way Analyses of Variance (ANOVAs) for the dependent variables. A Bonferroni test was also included to adjust probability (p) values due to the increased risk of a type I error when performing multiple statistical tests.

## 3. Results

Dependent variables included: activation intensity, duration, and frequency of facial expressions. Simple (AU12) and Social (AU12 and AU06) smiles were considered. Two series of Spearman′s rank-order correlations were conducted to understand the relationship between IQ, gender, and the dependent variables. No statistically significant correlation emerged (Table S1 in Supplementary Materials).

A one-way MANOVA was applied to test for statistically significant discrimination between TD and ASD groups, comparing features of two smile types. Preliminary assumption checking revealed that residuals were not normally distributed for some of the dependent variables, as assessed by Shapiro–Wilk’s test (*p* < 0.01). Despite the violation of the assumption, the MANOVA remains an optimal statistical model thanks to its robustness against deflections from normality. There were no multivariate outliers in the data, as assessed by Mahalanobis distance. There was no multicollinearity, as assessed by Pearson correlation (r = 0.91, *p* < 0.0001). Variances were homogenous, as assessed by Levene’s test (*p* > 0.05).

A statistically significant difference emerged between the groups on the combined dependent variables (ASD vs TD), (F (6, 22) = 14.513, *p* < 0.0001). Univariate one-way ANOVAs were performed as follow-up analyses, with a Bonferroni adjustment such that statistical significance was accepted at *p* < 0.00714 (Table S2 in Supplementary Materials).

**Figure 1:**
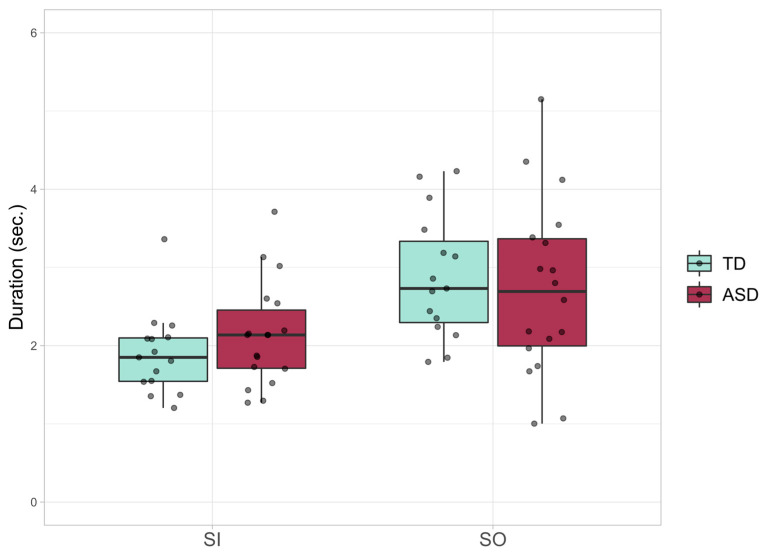
Smile Duration; boxplots of the average duration for Simple (SI) and Social (SO) Smiles in ASD (Autism Spectrum Disorder) and TD (typically developing) groups.

There were no statistically significant differences between the ASD and TD groups for duration of both Social (F(1, 27) = 0.219, *p* = 0.644, partial η2 = 0.008) and Simple Smiles (F(1, 27) = 0.992, *p* = 0.328, partial η2 = 0.035) (Figure 1).

**Figure 2:**
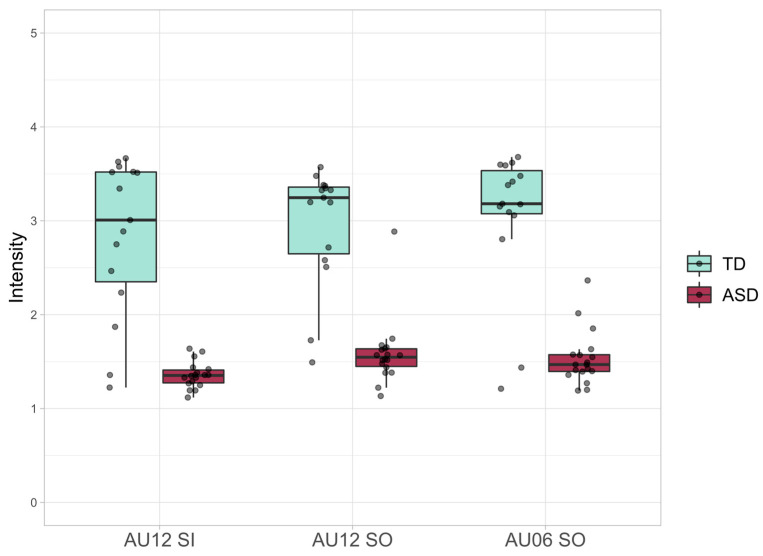
Smile Intensity; boxplots of Action Units 12 and 6 (AU12 and AU06) intensities for Social Smiles (SO) and Action Unit 12 (AU12) intensity for Simple Smiles (SI).

A statistically significant difference was found in adjusted means for activation intensity. In the case of Simple Smiles, the activation intensity of AU12 was significantly lower in the ASD group (F(1, 27) = 70.528, *p* < 0.0001, partial η2 = 0.723); in the Social smiles, both the AU06 (F(1, 27) = 80.293, *p* < 0.0001, partial η2 = 0.748) and the AU12 (F (1, 27) = 66.925, *p* < 0.0001, partial η2 = 0.713) were hypoactive in the ASD group (Figure 2).

**Figure 3:**
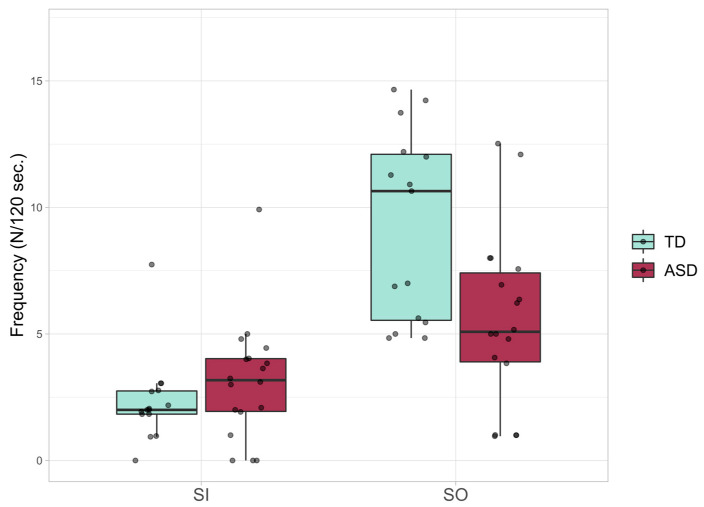
Smile Frequency; boxplots of the average frequency for Simple (SI) and Social (SO) Smiles in ASD and TD groups. Frequency is expressed in terms of the number of expressions over 120 s of interaction.

In terms of frequency, statistically significant differences emerged only in the case of Social Smiles (F(1, 27) = 11.526, *p* < 0.00714, partial η2 = 0.299), whereas the Simple Smiles frequency did not reveal a significant difference between the two groups (F(1, 27) = 3.133, *p* = 0.088, partial η2 = 0.104) (Figure 3).

Finally, pairwise comparisons with Bonferroni-adjusted *p*-values were made for all dependent variables (Table S3 in Supplementary Materials). Adjusted means were significantly different between groups in terms of activation intensity (*p* < 0.0001) for Simple and Social Smiles. Moreover, a significant difference in the adjusted mean for the frequency of Social Smiles was also found (*p* < 0.01). All other pairwise comparisons were not statistically significant.

## 4. Discussion

We applied computerized methods for human behavior analysis with the aim of exploring novel early markers of atypical socio-interactive development in infants with ASD. In this attempt, we have implemented a combination of Computer Vision and machine learning models for the refined analysis of facial dynamics through videos. The emphasis on attentive components in the processing of social stimuli has been highlighted in defining the early indicators of atypical neurodevelopment in ASD [13,30,34,35,36]. Social smiling is defined by the temporal embedding of a positive expressive component and the oriented gaze towards the face of another person [55]. In infancy, such facial expression is characterized by having a strong temporal link with the maturation of new orientation patterns towards the mother’s face [46,47,62]. Due to its expressive nature, the Social Smile has been suggested as a possible early marker of later manifestation of the ASD phenotype [23,55]. Using automated measurements, we analyzed micro components of Social Smiles in early positive interactions between caregivers and infants within the first year of life. We compared both communicative and non-communicative smiles dynamics in infants with ASD and TD controls. For each of the two expressive types, we extracted duration, intensity, and frequency.

The findings seem to suggest that subjects with ASD produce positive facial expressions with significantly lower intensity compared to those with typical development during the first year of development. This reduced activation of facial movements (measured by AUs [54]) occurs both from smiles with a social value and from Non-Social Smiles, suggesting a general poor positive expressive production during the first social exchanges in ASD [13,15,18]. In our data, a difference was found in frequency between the two types of smiles. Indeed, while there is a reduced frequency of social smiling in children with ASD, Simple Smiles, which lack a communicative value, have a similar frequency between ASD and TD groups. This result seems to replicate previous findings of lower rates for social smiling but similar average rates for non-social smiling in infants with ASD [55].

Moreover, the expressive deficit does not seem to correlate with the general cognitive functioning level in our ASD sample. This result is consistent with previous studies that suggested a relationship between IQ and compensatory strategies to reduce the expressive deficit in ASD [78,79]. It is reasonable to expect that this compensatory pattern is not yet evident in infancy when cognitive functioning does not support the development of compensatory mechanisms. Finally, there was no difference among the average duration of both Social and Simple Smiles between subjects with typical development and ASD. Overall, the value of the results emerging from this study takes on significance in two research contexts: a context more linked to clinical aspects of the pathology and the study of atypical development; and a context more linked to the contribution of new methodological strategies for human behavior analysis.

In the clinical context, the findings seem to suggest that it could be interesting to include the quantification of expressive dynamics as a potential feature in the early detection of ASD. To our knowledge, this study is the first to highlight potential differences in positive expressive dynamics already within the first year of development in infants with ASD. Our findings seem to suggest that fine-grained analysis of social smiling carries salient cues for early detection of anomalies in social development already starting at 6 months of life. Furthermore, the difference that emerged in frequency potentially contributes to underlining the importance of the attention component in identifying the condition at the early stages of development. Compared to Simple Smiles, less charged with a communicative social value, Social Smiles displayed differently both in intensity and frequency. This discrepancy between social and non-Social Smile dynamics seems to corroborate previous research emphasizing the importance of the primary attentive components [13,15,30,34,35,36].

Although the nature of the data collected through this study is not sufficient to define conclusions about specific mechanisms underlying an atypical socio-cognitive development, it may have heuristic value, including speculations. One possibility is that the lack of use of such a powerful communication tool (Social Smile) could induce the toddler with ASD to miss the experience or feedback from effective interactive exchanges during the first months of development. Such atypical behavior could cause a reduction of social experiences in a critical period [47] for the development of social cognition. In this perspective, identifying the disease as soon as possible is of crucial importance to be able to anticipate the full phenotype manifestation and provide the infant with ASD the tools to help him or her to compensate for this socio-interactive deprivation [1,15,31,32].

The innovative contribution of this study is also linked to methodological aspects of the analysis. Digital approaches allow for high-quality data and analyses by relying on quantitative and objective measurements to categorize behaviors, which could be non-invasive and automated [42,80]. These features make the application of such models extremely promising in ASD research, in which discrete ecological measurements are essential. In literature, such technologies have been proposed as alternative measurement tools for behavior in infants with ASD, targeting many of the markers that had already been identified in the past through more traditional methods, such as attentional and motor deficits [13,18,21,22,25,30,34,35,36,37,38,39,40,78]. To summarize, the goal of this work was to test AI in bringing out new features. To our knowledge, this is the first attempt to explore new markers of ASD during the first social dyadic interactions based on smiling. A poor generic positive expression in infants with ASD has often been reported in the literature. Specifically, the Social Smile has already been proposed as a possible initial marker of the ASD phenotype, but starting from the second year of life when many interactive anomalies related to the pathology occur [15,21,55]. The limit of such approaches, we hypothesize, may lie in having analyzed facial dynamics through complex and unrefined systems, paying attention to macro components of the smiles, such as the integration between expression and gaze, without paying attention to the micro facial components. The use of automated systems for the behavior analysis through the image [48] allowed us to effectively extract refined features of the facial movements (Action Units), which typically require a demanding manual encoding by experts [54]. By exploiting these technologies, we were able to compute a quantitative and systematic measurement of smiles in very early interactions within the first year of development. Beyond the aspects of methodological innovation, the findings are consistent with past research reporting early anomalies in primary socio-attentional components in infants with ASD [13,30,34,35,36].

Moreover, this study offers the opportunity to once again underline the promising contribution that AI-based methods may offer to clinical research and practice. The use of automated technologies allowed the extraction of fine features and potentially uncovered subtle atypia in the development, hardly detectable with the naked eye [8,42]. In ASD research, considerable emphasis has been placed on searching for reliable early indicators able to predict the risk of a later diagnosis. The advantage is to start intervention as soon as possible and hopefully improve the outcome [4,5,55,81]. AI is well-suited for the quantification of behavioral markers. AI-based technology can further lead to a non-invasive, objective, and automatic tool for autism research [82]. In this perspective, AI-based solutions offer excellent opportunities to explore new subtle features, taking advantage of extracting systematic measurements in a non-intrusive way. In real-world scenarios, such tools may offer the possibility of effective support for therapists and individuals with ASD by giving fast feedback on behavior monitoring and potentially supporting the diagnostic and therapeutic process. Setting up a more direct collaboration between the clinic and research would help to overcome the gap and move towards a wider therapeutic perspective by providing concrete solutions [83]. Despite their high potential clinical relevance, AI-based systems still suffer from limited translational applications [83]. Still, it is necessary to implement solutions that fit naturalistic contexts to obtain generalizable results. Tailoring these technologies to more ecological scenarios would promote closer clinic-research collaboration by providing concrete solutions [83].

### Limitations

The present research also carries some relevant methodological limitations. Home Videos provide the huge benefit of being able to have early ecological data, but at the expense of data control. The study has intentionally focused on retrospective material, intending to analyze features in a domestic and natural context. The literature suggests investigating facial expressions in as spontaneous environments as possible [74]. However, the more unconstrained the interactions, the less control over the data. To manage these limitations, we adopted strict selection rules for interactions. Still, a certain degree of data variability remains inevitable. In particular, including only play interactions meant it was not possible to control parental behavior, such as responsiveness and intensity of engagement. From a point of view more related to the characteristics of the population, also due to the characteristics of typically unstructured HVs, the total size of the two samples and the average number of interactions analyzed per subject is limited, reducing the explanatory power of the results. For these reasons, although the findings of this study appear to be promising, the results should be interpreted as exploratory. In the future, it would be interesting to replicate these analyses by involving larger samples of infants at risk for ASD, thus early monitoring of smiling in more structured contexts through interaction coding.

## 5. Conclusions

Timing is a major concern in the diagnosis of ASD. To achieve treatment effectiveness, the condition must be detected as soon as possible. In this study, the combination of Machine Learning models and a systematic approach uncovered new subtle indicators. Facial dynamics seem to play a salient role in this perspective, consistent with what has already been found in the literature. This study’s contribution is towards exposing novel early markers of ASD through behavior imaging. Moreover, the findings highlight the potential of expressiveness as a target for early intervention and the benefit of AI as a valuable support source for clinicians and family members in the detection of ASD.

## Figures and Tables

**Table 1:** Population characteristics of TD and ASD groups.

Variable	ASD	TD	*t/χ2*	*p*
	*n* = 18	*n* = 15		
Gender, *N* (%)			0.533	0.465
Male	17 (94.4)	13 (86.7)		
Female	1 (5.6)	2 (13.3)		
Age (months), mean (SD)	74.4 (41.5)	84.5 (29.5)	0.544	0.593
Average Video Age (months), mean (SD)	8.3 (1.2)	8.8 (1.7)	1.153	0.258
ADOS CSS Total Score, mean (SD)	7 (1.8)	−	−	−
IQ Composite Score, mean (SD)	76.2 (22.5) ^a^	95.8 (6.1) ^b^	−	−
Video Length (seconds), mean (SD)	121.7 (5.9)	123.3 (6.3)	0.723	0.475
Average Interactions number, mean (SD)	3.6 (0.7)	3.3 (0.8)	0.839	0.408
Confidence Score, mean (SD)	0.94 (0.01)	0.95 (0.14)	1.933	0.062
Confidence Percentage, mean (SD)	90.5 (5.8)	88.5 (8.3)	0.811	0.424

ADOS CSS: Autism Diagnostic Observation Schedule, 2nd edition, comparison score; IQ: Intelligence Quotient; ASD: Autism Spectrum Disorder; TD: Typically Developing. Average Video Age refers to the average age of subjects in the videos. Age refers to the age at the moment of evaluation. Average interaction number refers to the average number of interactions for each subject. Confidence Score refers to the average confidence (expressed from 0 to 1) of frames analyzed by Openface. Confidence Percentage refers to the proportion of frames with a high Confidence Score (>0.75) over the whole interaction video. IQ and ADOS scores were available only for the ASD group. ^a^ The average IQ score is calculated over 16 subjects from the ASD group. ^b^ The average IQ score is calculated over 4 subjects from the TD group.

## Data Availability

The parents of all the participants gave their consent to the publication of the results of the study, anonymously, in aggregated form. Due to ethical and privacy issues, sensitive data cannot be shared.

## Supplementary Materials

The following are available online at https://www.mdpi.com/article/10.3390/jcm10081776/s1, Table S1: Correlations between IQ, Gender and all dependent variables in the ASD group; Table S2: Results of follow up Multiple Comparisons; Table S3: Results of Pairwise Comparisons.

## Abbreviations

The following abbreviations are used in this manuscript:

ASD	Autism Spectrum Disorder
TD	Typically Developing
AI	Artificial Intelligence
CV	Computer Vision
CNN	Convolutional Neural Network
AU	Action Unit
FACS	Facial Action Coding System
H	Home Video
S	Social Smile
SI	Simple Smile
