# Supplementary material for: Is Smiling the Key? Machine Learning Analytics Detect Subtle Patterns in Micro-Expressions of Infants with ASD
Source: J Clin Med. 2021 Apr 19;10(8):1776. doi: 10.3390/jcm10081776 (PMC8073678; doi:10.3390/jcm10081776)
Supplement: Supplementary file 1 [file jcm-10-01776-s001.zip › jcm-1167282-supplementary.pdf]

Supplementary Materials

Table S1. Correlations between IQ, Gender and all dependent variables in the ASD group.

Variable		IQ	Gender
SO Duration (sec.)	rho	0.187	-0.215
	p value	0.458	0.39
SI Duration (sec.)	rho	-0.145	-0.158
	p value	0.955	0.531
SO Frequency	rho	0.336	-0.115
	p value	0.173	0.649
SI Frequency	rho	-0.361	-0.101
	p value	0.142	0.691
SO AU12 Intensity	rho	0.383	-0.33
	p value	0.116	0.181
SO AU06 Intensity	rho	0.363	-0.445
	p value	0.139	0.064
SI AU12 intensity	rho	0.141	-0.417
	p value	0.578	0.085

Note: SO Social Smile; SI Simple Smile; AU12, AU06 Action Unit 12, 6

Spearman's rank-order correlations
p<0.01;*p<0.001;****p<0.0001

Table S2. Results of follow up Multiple Comparisons.

Variable	ASD	TD	F	p	Effect Size
	Mean (SD)	Mean (SD)			
SO Duration (sec.)	81.6 (33.6)	86.1 (23.6)	0.219	0.644	0.008
SI Duration (sec.)	63.9 (21.8)	56.7 (16.4)	0.992	0.328	0.035
SO Frequency	5.5 (3.4)	9.3 (3.7)	11.526	<0.01**a	0.299
SI Frequency	3.1 (2.4)	2.3 (1.7)	3.133	0.088	0.104
SO AU12 Intensity	1.6 (0.4)	3.0 (0.6)	66.925	<0.0001****a	0.713
SO AU6 Intensity	1.5 (0.3)	2.9 (0.8)	80.293	<0.0001****a	0.748
SI AU12 intensity	1.4 (0.2)	2.9 (0.8)	70.528	<0.0001****a	0.723

Note: SO Social Smile; SI Simple Smile; AU12, AU06 Action Unit 12, 6
One-way Analysis of Variance; Effect sizes expressed as Eta-squared (η^2);

a Denotes comparisons that remained significant following Bonferroni correction.
p<0.01;*p<0.001;****p<0.0001

Table S3. Results of Pairwise Comparisons

Variable	conf. high	p adj.
SO Duration (sec.)	18.368	0.644
SI Duration (sec.)	21.982	0.328
SO Frequency	-1.753	<0.01** a
SI Frequency	2.655	0.088
SO AU12 Intensity	-1.091	<0.0001**** a
SO AU6 Intensity	-1.251	<0.0001**** a
SI AU12 intensity	0.463	<0.0001**** a

Note: SO Social Smile; SI Simple Smile; AU12, AU06 Action Unit 12, 6

Tukey's Honestly Significant Difference Test
a Denotes comparisons that remained significant following Bonferroni correction.
p<0.01;*p<0.001;****p<0.0001
